# Increasing prevalence, molecular characterization and antifungal drug susceptibility of serial *Candida auris* isolates in Kuwait

**DOI:** 10.1371/journal.pone.0195743

**Published:** 2018-04-09

**Authors:** Ziauddin Khan, Suhail Ahmad, Noura Al-Sweih, Leena Joseph, Wadha Alfouzan, Mohammad Asadzadeh

**Affiliations:** Department of Microbiology, Faculty of Medicine, Kuwait University, Safat, Kuwait; Institute of Microbiology, SWITZERLAND

## Abstract

*Candida auris* is an emerging yeast pathogen of global significance. Its multidrug-resistant nature and inadequacies of conventional identification systems pose diagnostic and therapeutic challenges. This study investigated occurrence of *C*. *auris* in clinical specimens in Kuwait and its susceptibility to antifungal agents. Clinical yeast strains isolated during 3.5-year period and forming pink-colored colonies on CHROMagar Candida were studied by wet mount examination for microscopic morphology and Vitek 2 yeast identification system. A simple species-specific PCR assay was developed for molecular identification and results were confirmed by PCR-sequencing of rDNA. Antifungal susceptibility testing of one isolate from each patient was determined by Etest. The 280 isolates forming pink-colored colonies on CHROMagar Candida, were identified by Vitek 2 as *Candida haemulonii* (n = 166), *Candida utilis* (n = 49), *Candida kefyr* (n = 45), *Candida guilliermondii* (n = 9), *Candida famata* (n = 6) and *Candida conglobata* (n = 5). Species-specific PCR and PCR-sequencing of rDNA identified 166 *C*. *haemulonii* isolates as *C*. *auris* (n = 158), *C*. *haemulonii* (n = 6) and *Candida duobushaemulonii* (n = 2). *C*. *auris* isolates originated from diverse clinical specimens from 56 patients. Of 56 *C*. *auris* isolates tested, all were resistant to fluconazole, 41/56 (73%) and 13/56 (23%) were additionally resistant to voriconazole and amphotericin B, respectively. Eleven (20%) isolates were resistant to fluconazole, voriconazole and amphotericin B. One isolate was resistant to caspofungin and micafungin. Increasing isolation of *C*. *auris* in recent years from diverse clinical specimens including bloodstream shows that *C*. *auris* is an emerging non-*albicans Candida* species in Kuwait causing a variety of infections. Inability of conventional identification methods to accurately identify this pathogen and multidrug-resistant nature of many strains calls for a greater understanding of its epidemiology, risk factors for acquiring *C*. *auris* infection and management strategies in high-risk patients. This is the first comprehensive study on the emergence of this multidrug-resistant yeast from Kuwait and the Middle East.

## Introduction

*Candida auris* is an emerging multidrug-resistant yeast that can cause invasive infections and is associated with high mortality [1–4]. The species was first identified in 2009 from external ear sample of a Japanese patient [5]. *C*. *auris* has now been recognized as an important nosocomial pathogen in many countries causing bloodstream infections, ventriculitis, osteomyelitis, otomastoiditis, chronic ostitis media, intraabdominal infections, pericarditis, pleural efffusion and vulvovaginitis [1, 3–8]. Outbreaks of *C*. *auris* infections have also been reported in hospitalized patients from Pakistan, the United Kingdom, Spain and Venezuela [2, 9–11]. *C*. *auris* is usually misidentified by routinely used phenotypic methods in clinical microbiology laboratories and many strains are intrinsically resistant to multiple antifungal agents [3, 7, 12, 13]. Due to high mortality rates associated with *C*. *auris* infections in critically ill patients, this species is now attracting worldwide attention as an opportunistic fungal pathogen [3, 7, 14]. Application of molecular identification methods based on sequencing of rDNA and/or protein profiling by Matrix-assisted laser desorption/ionization time-of-flight mass spectrometry (MALDI-TOF MS) have led to the recognition of *C*. *auris* infections in many countries of Africa, Europe, North and South Americas and Asia including the Middle East [1, 3, 13, 15]. Although all unusual and rare yeast isolates that were referred to the Mycology Reference Laboratory from 2005 were identified by PCR-sequencing of ITS region and/or D1/D2 domains of rDNA, no *C*. *auris* was identified until 2014. However, 7 *C*. *haemulonii* strains that caused an outbreak in Maternity Hospital in Kuwait were identified in 2005–2006 [16]. This retrospective study investigated the occurrence of *C*. *auris* among clinical yeast isolates forming pink-colored colonies on CHROMagar Candida that were recovered from diverse clinical specimens including bloodstream during a 3.5-year duration in Kuwait. A simple species-specific PCR assay was developed for rapid molecular identification of *C*. *auris* isolates and the results were confirmed by PCR-sequencing of rDNA. Antifungal susceptibility testing of one isolate from each patient was also determined.

## Materials and methods

### Reference strains, clinical isolates and phenotypic identification

Reference strains or well characterized clinical isolates of *Candida dubliniensis* (CD36), *Candida albicans* (ATCC 90028), *Candida glabrata* (ATCC 90030), *C*. *parapsilosis* (ATCC 22019), *C*. *orthopsilosis* (ATCC 96139), *Candida tropicalis* (ATCC 750), *Candida kefyr* (Kw1609/11), *Candida conglobata* (Kw381/16), *Candida utilis* (Kw3642/15), *Candida fermentati* (Kw3414/13), *Candida guilliermondii* (Kw14/14), *Candida haemulonii* (Kw154/06), *Candida duobushaemulonii* (Kw3270/08) and *Candida auris* (Kw2611/17) were used as reference *Candida* species. Clinical yeast strains cultured from various clinical specimens during a 3.5-year (May 2014 to September 2017) period forming pink-colored colonies on CHROMagar Candida and not recognized as one of the common *Candida* spp. by VITEK 2 were subjected to detailed phenotypic and molecular identification. The clinical specimens (including invasive samples) were collected from patients after obtaining verbal consent at different hospitals across Kuwait as part of routine diagnostic work-up for fungal infections. A total of 280 clinical yeast isolates forming pink-colored colonies on CHROMagar Candida were used. The isolates were sent from different hospitals across Kuwait to Mycology Reference Laboratory, Department of Microbiology, Faculty of Medicine; Kuwait University for identification and antifungal susceptibility testing. The isolates were studied by wet mount examination of the culture for microscopic morphology and by Vitek 2 yeast identification system (bioMérieux, Marcy-l’Etoile, France) for species identification. The study was approved by the Committee for the Protection of Human Subjects in Research, Health Sciences Center, Kuwait University and Ministry of Health, Kuwait (Approval no. VDR/EC/2477).

### Molecular identification and characterization

The genomic DNA from reference strains and clinical isolates of *Candida* species was prepared by the rapid method using Chelex-100, as described previously [17]. A simple, low-cost PCR assay was developed for rapid molecular identification of *C*. *auris* isolates. For this purpose, one forward (CAURF, 5’-ATTTTGCATACACACTGATTTG-3’) and one reverse (CAURR, 5’-CGTGCAAGCTGTAATTTTGTGA-3’) primer targeting specific sequences within ITS-1 and ITS-2 regions of rDNA of *C*. *auris* were synthesized. The unique primer sequences designed in this study were based on sequence alignment of ITS region sequences from multiple strains of all commonly encountered clinical yeast species that are available from the GenBank. The species specificity of the primers CAURF and CAURR for *C*. *auris* was indicated by BLAST searches (http://www.ncbi.nlm.nih.gov/entrez/query.fcgi) as they showed complete sequence identity only with *C*. *auris* strains. The reaction and PCR cycling conditions were same as described previously except that primers CAURF and CAURR were used [17]. PCR amplicons were run on 2% (w/v) agarose gels, as described previously [18]. The results of species-specific identification of 16 selected *C*. *auris* isolates and all *C*. *haemulonii/C*. *duobushaemulonii* isolates were confirmed by DNA sequencing of the ITS region of rDNA. The ITS region was amplified by using ITS1 and ITS4 panfungal primers and both strands of the amplicons were sequenced by using ITS1FS, ITS2, ITS3 and ITS4RS primers as described previously [19, 20]. BLAST searches (http://blast.ncbi.nlm.nih.gov/Blast.cgi?) were performed and >99% sequence identity was used for species identification.

The genotypic relationship among *C*. *auris* was studied by comparing ITS region sequences of *C*. *auris* isolates. Genotypic heterogeneity was further investigated by fingerprinting of the isolates by using minisatellite-based (M13-MIN, 5’-GAGGGTGGCGGTTCT-3’) and microsatellite-based (GACA_4_, 5’-GACAGACAGACAGACA-3’) primers, as described previously [21].

### Antifungal susceptibility testing

Antifungal susceptibility of 56 *C*. *auris* isolates (representing one isolate from each patient) was determined by Etest as described previously [22]. Etest strips for amphotericin B (AP), fluconazole (FL), voriconazole (VO) caspofungin (CS) and micafungin (MYC) (bioMérieux SA, Marcy-l’-Etoile, France) were used. So far, there are no approved antifungal susceptibility breakpoints for *C*. *auris*. However, based on CLSI and EUCAST susceptibility data, tentative minimum inhibitory concentration (MIC) breakpoints defining resistance have been proposed as follows: fluconazole, ≥32 μg/ml; voriconazole, ≥1 μg/ml; amphotericin B, ≥2 μg/ml; micafungin, ≥4 μg/ml and caspofungin, ≥2 μg/ml [1, 12, 23].

## Results

### Phenotypic and molecular identification of pink-colored colonies on CHROMagar Candida

A total of 280 isolates formed pink-colored colonies on CHROMagar Candida during the study period and were subjected to detailed phenotypic and molecular characterization. The Vitek 2 yeast identification system identified these isolates as *C*. *haemulonii* (n = 166), *Candida utilis* (n = 49), *Candida kefyr* (n = 45), *Candida guilliermondii* (n = 9), *Candida famata* (n = 6) and *Candida conglobata* (n = 5) (Table 1).

**Table 1:** Identification of 280 *Candida* spp. isolates forming pink-colored colonies on CHROMagar Candida by Vitek 2, *C*. *auris*-specifc PCR assay developed in this study and PCR-sequencing of rDNA.

Identification method	No. of isolates identified as								
	*C*. *haemulonii*	*C*. *auris*	*C*. *duobushaemulonii*	*C*. *utilis*	*C*. *kefyr*	*C*. *guilliermondii*	*C*. *famata*	*C*. *fermentati*	*C*. *conglobata*
Vitek 2	166	0	0	49	45	9	6^b^	0	5
*C*. *auris*-specific PCR	0	158	0	0	0	0	0	0	0
rDNA sequencing	6	16^a^	2	49	12^a^	4^a^	0	6^b^	5

^a^Only selected isolates for these species were analyzed by PCR-sequencing of rDNA

^b^All six isolates identified as *C*. *famata* were correctly identified as *C*. *fermentati* by PCR-sequencing of rDNA

The PCR amplification performed with CAURF and CAURR primers yielded an expected size amplicon of nearly 276 bp with DNA extracted from the reference strain of *C*. *auris* only while no amplicon was obtained with genomic DNA prepared from reference strains or well characterized clinical isolates of *C*. *dubliniensis*, *C*. *albicans*, *C*. *glabrata*, *C*. *parapsilosis*, *C*. *orthopsilosis*, *C*. *tropicalis*, *C*. *kefyr*, *C*. *utilis*, *C*. *guilliermondii*, *C*. *haemulonii*, and *C*. *duobushaemulonii*, as expected (S1 Fig). No amplicon was also obtained with DNA isolated from *C*. *fermentati* as well as with human DNA (data not shown). Of 280 isolates analyzed in this study, no amplicon was obtained during PCR amplification with CAURF and CAURR primers with DNA isolated from 114 isolates forming pink-colored colonies on CHROMagar Candida and identified as *C*. *utilis* (n = 49), *C*. *kefyr* (n = 45), *C*. *guilliermondii* (n = 9), *C*. *famata* (n = 6) and *C*. *conglobata* (n = 5) by Vitek 2. However, PCR amplification of DNA isolated from 166 isolates phenotypically identified as *C*. *haemulonii* (described above) by Vitek 2 with CAURF and CAURR primers showed that 158 isolates were actually *C*. *auris* as they yielded an amplicon of 276 bp while the remaining 8 isolates did not yield an amplicon (Table 1). PCR-sequencing of 16 selected *C*. *auris* isolates matched completely with the ITS region sequence of reference and several other strains of *C*. *auris* from India but were different from the sequences of *C*. *auris* isolates from Japan, South Africa and Venezuela. PCR-sequencing of ITS region of rDNA also identified the latter 8 isolates as *C*. *haemulonii* (n = 6) and *C*. *duobushaemulonii* (n = 2) (Table 1). PCR-sequencing of rDNA also identified all *C*. *utilis*, *C*. *conglobata* and selected isolates of *C*. *kefyr* and *C*. *guilliermondii*. However, all 6 isolates identified as *C*. *famata* by Vitek 2 were correctly identified as *C*. *fermentati* by PCR-sequencing of rDNA (Table 1). None of the *C*. *auris* isolate showed hyphae or pseudohyphae on wet mount examination of cultures [3].

### Epidemiology of *C*. *auris* infections in Kuwait

The 158 *C*. *auris* isolates came from 56 (31 males and 25 females) patients (S1 Table). All the patients yielding *C*. *auris* were admitted to intensive care units (ICUs) or other wards in different hospitals across Kuwait for various life-threatening conditions and for different time-periods extending up to several months in some cases. The patient’s age ranged from 13 to 89 years. None of the *C*. *auris* isolate came from a pediatric patient (≤12 years). Thirty-five patients were Kuwaiti nationals and 10 patients were expatriates originating from 5 different countries while the remaining 11 were only classified as non-Kuwaiti patients. The origin of 158 *C*. *auris* isolates from different specimens is provided in Table 2. Thirty-one patients yielded multiple isolates either from the same or different clinical specimens and seven patients remained colonized with *C*. *auris* for an extended period of time ranging from 70 days to 11 months (S1 Table). Interestingly, 16 bloodstream isolates were obtained from 13 patients and 5 of these patients also yielded *C*. *auris* from 1–4 other anatomical sites (S1 Table). Furthermore, 24 elderly (≥65 year old) patients yielded most (83 of 158, 53%) of the *C*. *auris* isolates described in this study and 21 of these patients were hospitalized in a single healthcare facility. Also, 12 patients were ≥80 year old, hospitalized in the same facility and yielded 60 isolates from different/multiple anatomic sites (S1 Table).

**Table 2:** Origin of 158 *C*. *auris* isolates in diverse clinical specimens obtained from 56 patients in Kuwait.

Specimen/source	No. of patients*	No. of isolates (%)
Blood	13	16 (9.5)
Urine	27	53 (33.5)
Tracheal aspirate	21	51 (32.3)
Catheter tip	5	8 (5.1)
Sputum	6	9 (5.1)
Pus/wound swab	2	5 (3.2)
Vaginal swab	4	4 (2.5)
Abdominal fluid	2	3 (1.9)
Pleural fluid	2	2 (1.3)
Arteriovenous fistula tissue	1	2 (1.3)
Bronchoalveolar lavage	1	1 (0.6)
PEG tube swab	1	1 (0.6)
Eye swab	1	1 (0.6)
Nasal swab	1	1 (0.6)
Unidentified	1	1 (0.6)

PEG tube swab—Percutaneous endoscopic gastrostomy tube swab,

*Some patients yielded isolates from more than one anatomical site.

### Molecular fingerprinting of *C*. *auris* isolates

The ITS region of all 16 *C*. *auris* isolates that were sequenced were identical. Furthermore, PCR fingerprinting performed with minisatellite-based (M13-MIN) and microsatellite-based (GACA_4_) primers yielded identical patterns of amplified fragments from all *C*. *auris* isolates (data from 6 selected isolates are shown in S2 Fig) suggesting close genotypic relationship among the isolates.

### Antifungal susceptibility testing data

Antifungal susceptibility of *C*. *auris* was determined for one representative isolate from each patient and the data are presented in Table 3. All isolates were resistant to fluconazole with MICs of 128 to ≥256 μg/ml. The MICs for voriconazole were quite variable ranging between 0.064 to 6 μg/ml (geometric mean 1.20±1.06 μg/ml). Taking an arbitrary break point of ≥1 μg/ml, 41 (73.2%) isolates were additionally resistant to voriconazole. Resistance to amphotericin B (≥2 μg/ml) was observed in 13 (23.2%) isolates with a geometric mean of 1.05±0.74 μg/ml (Table 3). Eleven (19.6%) isolates were resistant to fluconazole, voriconazole and amphotericin B. One isolate obtained from urine specimen of a patient who was operated for colorectal cancer was resistant to caspofungin and micafungin (MIC = 4 μg/ml). The MIC50 and MIC90 values for amphotericin B, fluconazole, voriconazole, caspofungin and micafungin were 1.5 and 2 μg/ml, ≥256 and ≥256 μg/ml, 1.5 and 3 μg/ml, 0.19 and 0.38 μg/ml and 0.094 and 0.125 μg/ml, respectively (Table 3).

**Table 3:** MIC50, MIC90, MIC range and geometric mean of five antifungal agents for 56 *C*. *auris* isolates by Etest.

Antifungals	MIC50	MIC90	Range	GM ± SD	% Resistance*
Amphotericin (AP)	1.5	2	0.047–3	1.05 ± 0.74	23.21
Fluconazole (FL)	≥256	≥256	128-≥256	252.8 ± 17.25	100
Voriconazole (VO)	1.5	3	0.064–6	1.20 ± 1.06	73.2
Caspofungin (CS)	0.19	0.38	0.012–4	0.19 ± 0.53	1.8
Micafungin (MYC)	0.094	0.125	0.006–4	0.093 ± 0.524	1.8

GM, Geometric mean; SD, standard deviation

*Tentative MIC breakpoints: amphotericin B (AP) ≥ 2μg/ml; fluconazole (FL) ≥ 32μg/ml;

voriconazole (VO) ≥ 1 μg/ml; caspofungin (CS) ≥ 2μg/ml; micafungin (MYC) ≥ 4 μg/ml.

## Discussion

Colony characteristics (pink-colored colonies) of *C*. *auris* on CHROMagar Candida are similar to several other *Candida* species and commercial yeast identification systems such as Vitek 2 also routinely identify clinical *C*. *auris* isolates as *C*. *haemulonii* [3, 13, 24, 25]. With the application of molecular methods in clinical microbiology laboratories and some other, as yet, undefined factors, this multidrug-resistant species is now being isolated from clinical specimens with increasing frequency worldwide [2, 9–11, 14, 26–32]. *C*. *auris* was isolated for the first time in May 2014 in Kuwait from a case of fungemia in a 27-year-old woman who was admitted to intensive care unit for septic shock secondary to lobar pneumonia [33]. During the same year, 4 additional isolates were recovered from urine specimens of three individual Kuwaiti patients (including 2 isolates from one patient) admitted in the same hospital. This is despite the fact that all yeast isolates not belonging to common *Candida* species were identified by PCR-sequencing of ITS region and/or D1/D2 domains of rDNA from 2005 in Kuwait [34–37]. This approach was successful in identifying 7 *C*. *haemulonii* isolates that caused an outbreak in Maternity hospital in 2005–2006 [16]. The frequency of isolation of *C*. *auris* increased substantially after 2014 as 37 isolates were cultured during 2015, 60 isolates were cultured in 2016 and 56 isolates were cultured until September 2017. Furthermore, 3 and 4 bloodstream isolates were obtained from 2 and 3 candidemia patients in 2015 and 2016, respectively. Consistent with this increasing trend of *C*. *auris* infections, 8 bloodstream isolates from 7 candidemia patients were already recovered in the first 9 months of 2017. In addition to bloodstream, *C*. *auris* was also isolated from tissue specimens obtained from arteriovenous fistula, abdominal fluid/pleural fluid specimens and eye swab from a case of endophthalmitis suggesting invasive infections (S1 Table). Interestingly, 24 elderly patients yielded 83 of 158 (53%) *C*. *auris* isolates from different/multiple specimens and were colonized for extended period of time in some cases. Notably, 21 of 24 elderly patients (including all ≥80 year-old patients) with protracted illness were hospitalized in a single healthcare facility, suggesting the presence/persistence of *C*. *auris* in the indoor environment of this hospital. The persistence of *C*. *auris* in the hospital environment has also been noted in several other studies (7, 9, 10, 31).

All 56 individual patient *C*. *auris* isolates were resistant to fluconazole, 13 (23%) were resistant to fluconazole and amphotericin B and 11 (19.6%) were resistant to fluconazole, voriconazole and amphotericin B. No obvious reason could be attributed to the sudden increase in the isolation of this multidrug-resistant pathogen in Kuwait in recent years. Similar observations of rapid emergence of *C*. *auris* in some states of USA, where molecular detection of fungal pathogens is routinely performed, have also been made [14, 32, 33]. Of 174 confirmed clinical *C*. *auris* cases reported from 10 states in USA until November 30, 2017, 148 cases were reported from only two states on the east coast (New York, n = 110 and New Jersey, n = 38) showing a disproportionate distribution of these cases in different states in USA (https://www.cdc.gov/fungal/diseases/candidiasis/tracking-c-auris.html). The reasons for this preponderance of *C*. *auris* cases in these two states are not clear. Furthermore, 257 patients in only 4 states with clinical cases were found to be colonized with *C*. *auris*. A large outbreak of *C*. *auris* infections involving 44 patients has also been reported in hospitalized patients from the United Kingdom highlighting the rapid emergence and spreading of this multidrug-resistant pathogen in healthcare facilities in this country [9]. Another large outbreak involving 64 candidemia cases in Valencia, Spain has also been described recently [Ruiz-Gaitan, unpublished data].

The ability of *C*. *auris* isolates to grow at 40–42°C, inability to produce hyphae or pseudohyphae in culture and resistance to fluconazole should prompt clinical microbiology laboratories to seek identification of the yeast isolate by molecular methods [3, 24, 25, 38]. In a recent study, growth characteristics of four *C*. *auris* isolates with other *Candida* species in Sabouraud broth and yeast nitrogen base containing 10% NaCl (wt/vol) incubated at 40°C with/without supplementation with dextrose, dulcitol or mannitol were compared [39]. While *C*. *auris* strains grew in this medium, *C*. *haemulonii*, *C*. *doubushaemulonii*, *C*. *albicans* and *C*. *parapsilosis* isolates failed to grow. *C*. *glabrata* showed growth only in Sabouraud broth containing dextrose [39]. Recently, CHROMagar Candida medium supplemented with Pal’s medium has been evaluated for differentiation of *C*. *auris* from isolates belonging to *C*. *haemulonii* complex [40]. However, large-scale evaluation of this medium is required for reliable identification of *C*. *auris*.

For rapid molecular identification, Kordalewska et al. [15] developed a species-specific thermal PCR and a *C*. *haemulonii* complex-specific real-time PCR assay to identify *C*. *auris* and related species (*C*. *haemulonii*, *C*. *duobushaemulonii* and *C*. *lusitaniae*). The species-specific PCR assay developed in this study for rapid identification of *C*. *auris* is also simple and cost effective as the whole procedure can be completed within 4 hours using basic PCR and gel electrophoresis equipment and will cost only ~1 US$ per sample (excluding the cost of culture and personnel time). MALDI-TOF MS can also differentiate *C*. *auris* from other *Candida* species [24, 25]. We used 12 other *Candida* species during specificity testing of our PCR assay. The DNA from *C*. *fermentati* also did not yield an amplicon in PCR, as expected (data not shown). However, we did not test *C*. *famata*, *R*. *glutinis* and *C*. *catenulata* which are sometimes reported in place of *C*. *auris* by Vitek 2. However, based on BLAST searches, our primers do not show identity with the sequences of the corresponding region from these species. Although updated versions of MALDI-TOF MS can identify *C*. *auris*, it is yet to be updated in Kuwait. PCR-sequencing of 16 selected *C*. *auris* isolates were identical and matched completely with corresponding sequences of *C*. *auris* from India but were different from the sequences of *C*. *auris* isolates from Japan, South Africa and Venezuela. All *C*. *auris* isolates also yielded identical fingerprinting patterns by minisatellite-based and microsatellite-based primers which is consistent with the highly clonal nature of this yeast species [2, 7, 27].

Consistent with published reports [2, 3, 24], all (56, 100%) individual patient’s *C*. *auris* isolates were resistant to fluconazole and 41 (73.2%) isolates to voriconazole. Also 13 (23%) isolates were resistant to amphotericin B highlighting the multidrug-resistant (MDR) nature of this organism (1–4, 7, 14, 24, 26, 27). Only one isolate in our study was resistant to caspofungin and micafungin (MIC = 4 μg/ml). This isolate was obtained from the urine specimen of a patient who was operated for colorectal cancer. Resistance to echinocandins in *Candida* species is generally low, not exceeding 3% [41, 42]. Resistance of *C*. *auris* to echinocandins has also been found to be generally lower compared to polyenes [3, 27]. However, resistance in up to 7% *C*. *auris* isolates has been reported in one study [24].

Although 13 (23%) isolates were classified as resistant to amphotericin B, the actual number of amphotericin B-resistant isolates may be even higher. This is due to the fact that no clinical breakpoints are available for amphotericin B for *C*. *auris*. Tentative breakpoints for fluconazole, amphotericin B, and echinocandins have recently been proposed [2, 12, 21]. The Etest invariably yields much lower MIC values for amphotericin B (presumably due to a narrow calibration of concentration gradient on the strip). In a previous study comparing MIC distribution of 90 *C*. *auris* isolates by CLSI-BMD, Vitek 2 and Etest, the MIC_90_ values for amphotericin B were 4, 16 and 1 μg/ml, respectively [24]. Using tentative breakpoint of ≥2 μg/ml for amphotericin B resistance, the percentage of resistant isolates by the CLSI-BMD, Vitek 2 and Etest were 15.5%, 100% and 1.1%, respectively, indicating considerable differences in the MICs obtained by the three methods [24]. Based on pharmacokinetic studies in a mouse model of *C*. *auris* infection [43], it has been suggested that isolates showing MIC of ≥2 μg/ml by broth microdilution test may be considered as resistant to amphotericin B. Since under standard dosing, the breakpoints for amphotericin B are estimated to be 1 or 1.5 μg/ml, it may, therefore, be appropriate if Etest MICs of 1.5 μg/ml for *C*. *auris* are also rounded off to 2 μg/ml, as is practiced for other *Candida* species [21, 44]. If these revised cut-off values are adopted in our study, the number of isolates classified as amphotericin B-resistant will climb to 29 (52%).

*Candida auris* is a persistent colonizer and difficult to eradicate from the hospital environment [4, 6, 39, 45]. Its ability to form biofilms on a variety of surfaces and lack of activity of some antifungal agents such as fluconazole against *C*. *auris* may have contributed to its persistence and nosocomial transmission [3, 9, 30, 31, 39, 46]. In our study, two patients (both 80 year-old females) remained colonized for nearly 9 months (P39) and 11 months (P29) in the respiratory and/or urinary tract and *C*. *auris* was also isolated from the catheter tip, however, both patients did not develop candidemia, likely because they were treated with caspofungin prophylactically. In addition to demonstrating excellent activity of echinocandins against *C*. *auris*, our data also show that this multidrug-resistant species persists in colonized patients in hospital settings and the importance of devising effective measures for decontamination of the patients to prevent further inter- and intra-hospital transmission of *C*. *auris* [3, 4, 6, 47, 48].

Our study has a few limitations. i) The MICs for antifungal drugs were only determined by Etest and not by reference broth microdilution method. ii) The MIC values were determined for the first isolate from each patient and were not determined for repeat isolates.

## Conclusions

Isolation of *C*. *auris* from diverse clinical specimens including bloodstream with increasing frequency in recent years show that *C*. *auris* is a rapidly emerging pathogen in Kuwait. A simple, low-cost PCR assay has also been developed for its rapid identification. All *C*. *auris* isolates obtained from hospitalized patients in Kuwait were resistant to fluconazole, and 13 and 11 isolates were multidrug-resistant strains as they were additionally resistant to amphotericin B and amphotericin B + voriconazole, respectively. Inability of conventional yeast identification methods to accurately identify this pathogen and multidrug-resistant nature of many strains calls for a greater understanding of its epidemiology, risk factors for acquiring *C*. *auris* infection and management strategies in high-risk patients. This is the first comprehensive report highlighting the increasing prevalence of this multidrug-resistant yeast in Kuwait and the Middle East.

## Supporting information

S1 Table Source and date of isolation and demographic data of 56 patients yielding 158 *C*. *auris* isolates in Kuwait analyzed in this study. (DOCX)

S1 Fig Agarose gel of PCR amplicons obtained with *C*. *auris*-specific (CAURF and CAURR) primers and genomic DNA from reference strains of *C*. *dubliniensis* (lane 1), *C*. *albicans* (lane 2), *C*. *glabrata* (lane 3), *C*. *parapsilosis* (lane 3), *C*. *orthopsilosis* (lane 4), *C*. *tropicalis* (lane 5), *C*. *kefyr* (lane 6), *L*. *conglobata* (lane 7), *C*. *utilis* (lane 8), *C*. *guilliermondii* (lane 9), *C*. *haemulonii* (lane 10), *C*. *duobushaemulonii* (lane 11) and *C*. *auris* (lane 12). Lane M is 100 bp DNA ladder and the positions of migration of 100 bp, 300 bp and 600 bp fragments are marked.
(DOCX)

S2 Fig Agarose gel of PCR products obtained during fingerprinting with minisatellite-based (M13-MIN) primer (Panel A) and microsatellite-based (GACA_4_) primer (Panel B) with genomic DNA isolated from 6 *C*. *auris* isolates (lanes 1–6). Lane M is 100 bp DNA ladder and the positions of migration of 100 bp and 600 bp fragments are marked.
(DOCX)
