# Supplementary material for: Increasing prevalence, molecular characterization and antifungal drug susceptibility of serial Candida auris isolates in Kuwait
Source: PLoS One. 2018 Apr 9;13(4):e0195743. doi: 10.1371/journal.pone.0195743 (PMC5891028; doi:10.1371/journal.pone.0195743)
Supplement: S1 Table — (DOCX) [file pone.0195743.s001.docx]

**S1 Table**. **Source and date of isolation and demographic data of 56 patients yielding 158 *C. auris* isolates in Kuwait analyzed in this study.**

| Patient (P) | Clinical | Isolate | Date (dd.mm.yyyy) | Patient's demographic data | | |
| --- | --- | --- | --- | --- | --- | --- |
| no.^a^ | specimen | no. | of *C. auris* isolation | Age (years) | Gender^c^ | Nationality |
| 1 | Blood | Kw1732/14 | 26.05.2014 | 27 | F | Kuwaiti |
| 2 | Urine | Kw2857/14 | 18.09.2014 | 79 | F | Kuwaiti |
| 3 | Urine | Kw3361/14 | 05.11.2014 | 80 | M | Kuwaiti |
| **4** | Urine | Kw3716/14 | 27.11.2014 | 75 | F | Kuwaiti |
|  | Urine | Kw3971/14 | 21.12.2014 | 75 | F | Kuwaiti |
|  | Urine | Kw291/15 | 26.01.2015 | 75 | F | Kuwaiti |
|  | Urine | Kw348/15 | 29.01.2015 | 75 | F | Kuwaiti |
|  | Urine | Kw408/15 | 02.02.2015 | 75 | F | Kuwaiti |
|  | Urine | Kw636/15 | 17.02.2015 | 75 | F | Kuwaiti |
| 5 | Urine | Kw10/2015 | 04.01.2015 | 70 | M | Kuwaiti |
| 6 | Nasal swab | Kw147/15 | 13.01.2015 | 46 | F | Kuwaiti |
| 7 | Blood | Kw1192/15 | 07.04.2015 | N. A. | F | Filipino |
| 8 | Urine | Kw1548/15 | 12.05.2015 | 58 | F | Syrian |
| **9** | Wound swab | Kw2627/15 | 01.09.2015 | 45 | M | Pakistani |
|  | Wound swab | Kw3094/15 | 19.10.2015 | 45 | M | Pakistani |
|  | Tracheal aspirate | Kw3328/15 | 04.11.2015 | 45 | M | Pakistani |
| **10** | Urine | Kw2689/15 | 07.09.2015 | 85 | F | Kuwaiti |
|  | Catheter tip | Kw2690/15 | 07.09.2015 | 85 | F | Kuwaiti |
|  | Urine | Kw2762/15 | 14.09.2015 | 85 | F | Kuwaiti |
| 11 | Urine | Kw2864/15 | 28.09.2015 | 82 | F | Non-Kuwaiti^d^ |
| 12 | Urine | Kw2946/15 | 05.10.2015 | 43 | M | Syrian |
| 13 | Blood | Kw3391/15 | 11.11.2015 | 51 | M | Egyptian |
| **14** | Urine | Kw3420/15 | 15.11.2015 | 69 | M | Non-Kuwaiti |
|  | Urine | Kw3491/15 | 18.11.2015 | 69 | M | Non-Kuwaiti |
| **15** | Urine | Kw3626/15 | 01.12.2015 | 82 | F | Kuwaiti |
|  | Tracheal aspirate | Kw3627/15 | 01.12.2015 | 82 | F | Kuwaiti |
|  | Urine | Kw3666/15 | 06.12.2015 | 82 | F | Kuwaiti |
|  | Tracheal aspirate | Kw3758/15 | 13.12.2015 | 82 | F | Kuwaiti |
|  | Urine | Kw3824/15 | 16.12.2015 | 82 | F | Kuwaiti |
|  | Sputum | Kw3879/15 | 21.12.2015 | 82 | F | Kuwaiti |
|  | Sputum | Kw61/16 | 06.01.2016 | 82 | F | Kuwaiti |
|  | Tracheal aspirate | Kw849/16 | 14.03.2016 | 82 | F | Kuwaiti |
| 16 | Tracheal aspirate | Kw3734/15 | 10.12.2015 | 56 | M | Kuwaiti |
| **17** | PEG tube swab^b^ | Kw3803/15 | 15.12.2015 | 65 | M | Kuwaiti |
|  | Urine | Kw442/17 | 01.02.2017 | 65 | M | Kuwaiti |
|  | Urine | Kw1100-4-17 | 05.04.2017 | 65 | M | Kuwaiti |
| **18** | Tracheal aspirate | Kw3825/15 | 16.12.2015 | 82 | M | Kuwaiti |
|  | Urine | Kw3833/15 | 17.12.2015 | 82 | M | Kuwaiti |
|  | Blood | Kw3849/15 | 20.12.2015 | 82 | M | Kuwaiti |
|  | Blood | Kw3851/15 | 20.12.2015 | 82 | M | Kuwaiti |
|  | Tracheal aspirate | Kw3910/15 | 23.12.2015 | 82 | M | Kuwaiti |
|  | Urine | Kw3915/15 | 27.12.2015 | 82 | M | Kuwaiti |
| **19** | Vaginal swab | Kw3946/15 | 28.12.2015 | 75 | F | Kuwaiti |
|  | Urine | Kw4028/15 | 31.12.2015 | 75 | F | Kuwaiti |
|  | Tracheal aspirate | Kw6/16 | 04.01.2016 | 75 | F | Kuwaiti |
| 20 | Tracheal aspirate | Kw3997/15 | 29.12.2015 | 58 | M | Non-Kuwaiti |
| **21** | Tracheal aspirate | Kw4009/15 | 30/12/15 | 42 | M | Indian |
|  | Tracheal aspirate | Kw28/16 | 05.01.2016 | 42 | M | Indian |
| 22 | Sputum | Kw133/16 | 13.01.2016 | 53 | M | Kuwaiti |
| **23** | Urine | Kw322-1/16 | 27.01.2016 | 63 | F | Kuwaiti |
|  | Urine | Kw322-2/16 | 27.01.2016 | 63 | F | Kuwaiti |
| **24** | Sputum | Kw405/16 | 02.02.2016 | 46 | M | Indian |
|  | Tracheal aspirate | Kw541/16 | 15.02.2016 | 46 | M | Indian |
|  | Sputum | Kw809/16 | 09.03.2016 | 46 | M | Indian |
| **25** | Tracheal aspirate | Kw600/16 | 21.02.20/16 | 46 | M | Kuwaiti |
|  | Tracheal aspirate | Kw769/16 | 07.03.2016 | 46 | M | Kuwaiti |
| **26** | Sputum | Kw650/16 | 24.02.2016 | 24 | M | Kuwaiti |
|  | Sputum | Kw850/16 | 14.03.2016 | 24 | M | Kuwaiti |
| 27 | Unidentified | Kw1079/16 | 03.04.2016 | 69 | F | Kuwaiti |
| **28** | Urine | Kw1133/16 | 06.04.2016 | 89 | F | Kuwaiti |
|  | Vaginal swab | Kw1179/16 | 11.04.2016 | 89 | F | Kuwaiti |
|  | Tracheal aspirate | Kw1617/16 | 15.05.2016 | 89 | F | Kuwaiti |
| **29** | Catheter tip | Kw1501/16 | 04.05.2016 | 80 | F | Kuwaiti |
|  | Tracheal aspirate | Kw1927/16 | 12.06.2016 | 80 | F | Kuwaiti |
|  | Tracheal aspirate | Kw2075/16 | 26.06.2016 | 80 | F | Kuwaiti |
|  | Tracheal aspirate | Kw2233/16 | 14.07.2016 | 80 | F | Kuwaiti |
|  | Tracheal aspirate | Kw2140/16 | 03.07.2016 | 80 | F | Kuwaiti |
|  | Tracheal aspirate | Kw2454/16 | 11.08.2016 | 80 | F | Kuwaiti |
|  | Catheter tip | Kw2514/16 | 17.08.2016 | 80 | F | Kuwaiti |
|  | Tracheal aspirate | Kw2522/16 | 18.08.2016 | 80 | F | Kuwaiti |
|  | Catheter tip | Kw1056/17 | 02.04.2017 | 80 | F | Kuwaiti |
|  | Catheter tip | Kw1226/17 | 18.4.2017 | 80 | F | Kuwaiti |
| **30** | Tracheal aspirate | Kw1599/16 | 12.05.2016 | 44 | F | Kuwaiti |
|  | Urine | Kw1690/16 | 19.05.2016 | 44 | F | Kuwaiti |
|  | Tracheal aspirate | Kw1770/16 | 26.05.2016 | 44 | F | Kuwaiti |
|  | Urine | Kw2112/16 | 29.06.2016 | 44 | F | Kuwaiti |
| **31** | Blood | Kw1670/16 | 17.05.2016 | 46 | M | Kuwaiti |
|  | Blood | Kw1672/16 | 17.05.2016 | 46 | M | Kuwaiti |
| **32** | Tracheal aspirate | Kw1836/16 | 02.06.2016 | 85 | F | Kuwaiti |
|  | Tracheal aspirate | Kw2122/16 | 30.06.2016 | 85 | F | Kuwaiti |
|  | Urine | Kw2296/16 | 20.07.2016 | 85 | F | Kuwaiti |
| **33** | Abdominal fluid | Kw1979/16 | 15.06.2016 | 53 | F | Kuwaiti |
|  | Abdominal fluid | Kw2108/16 | 29.06.2016 | 53 | F | Kuwaiti |
|  | Blood | Kw2370/16 | 28.07.2016 | 53 | F | Kuwaiti |
| **34** | Urine | Kw1988/16 | 16.06.2016 | 51 | M | Non-Kuwaiti |
|  | Urine | Kw2165/16 | 10.07.2016 | 51 | M | Non-Kuwaiti |
|  | Urine | Kw2232/16 | 14.07.2016 | 51 | M | Non-Kuwaiti |
|  | Urine | Kw2267/16 | 18.07.2016 | 51 | M | Non-Kuwaiti |
|  | Urine | Kw2316/16 | 21.07.2016 | 51 | M | Non-Kuwaiti |
| **35** | Tissue | Kw1983/16 | 16.06.2016 | 52 | M | Non-Kuwaiti |
|  | Urine | Kw2007/16 | 19.06.2016 | 52 | M | Non-Kuwaiti |
|  | Wound swab | Kw2030/16 | 20.06.2016 | 52 | M | Non-Kuwaiti |
|  | Abdominal fluid | Kw2031/16 | 20.06.2016 | 52 | M | Non-Kuwaiti |
|  | Catheter tip | Kw2039/16 | 21.06.2016 | 52 | M | Non-Kuwaiti |
|  | Wound swab | Kw2178/16 | 10.07.2016 | 52 | M | Non-Kuwaiti |
|  | Pus swab | Kw2236/16 | 14.07.2016 | 52 | M | Non-Kuwaiti |
|  | Urine | Kw2253/16 | 17.07.2016 | 52 | M | Non-Kuwaiti |
|  | Tissue | Kw2254/16 | 17.07.2016 | 52 | M | Non-Kuwaiti |
| 36 | Eye swab | Kw2255/16 | 17.07.2016 | 65 | F | Egyptian |
| 37 | Blood | Kw2346/16 | 26.07.2016 | 65 | M | Non-Kuwaiti |
| 38 | Tracheal aspirate | Kw2385/16 | 02.08.2016 | 62 | F | Filipino |
| **39** | Urine | Kw2442/16 | 10.08.2016 | 80 | F | Kuwaiti |
|  | Tracheal aspirate | Kw2515/16 | 17.08.2016 | 80 | F | Kuwaiti |
|  | Urine | Kw2555/16 | 23.08.2016 | 80 | F | Kuwaiti |
|  | Urine | Kw2580/16 | 25.08.2016 | 80 | F | Kuwaiti |
|  | Tracheal aspirate | Kw2581/16 | 25.08.2016 | 80 | F | Kuwaiti |
|  | Tracheal aspirate | Kw369/17 | 26.01.2017 | 80 | F | Kuwaiti |
|  | Tracheal aspirate | Kw674/17 | 23.02.2017 | 80 | F | Kuwaiti |
|  | Vaginal swab | Kw716/17 | 28.02.2017 | 80 | F | Kuwaiti |
|  | Urine | Kw726/17 | 01.03.2017 | 80 | F | Kuwaiti |
|  | Urine | Kw1138/17 | 10.04.2017 | 80 | F | Kuwaiti |
|  | Catheter tip | Kw1217/17 | 17.04.2017 | 80 | F | Kuwaiti |
|  | Urine | Kw1225/17 | 18.04.2017 | 80 | F | Kuwaiti |
|  | Urine | Kw1239/17 | 19.04.2017 | 80 | F | Kuwaiti |
|  | Catheter tip | Kw1272/17 | 24.04.2017 | 80 | F | Kuwaiti |
|  | Urine | Kw1308/17 | 26.04.2017 | 80 | F | Kuwaiti |
|  | Tracheal aspirate | Kw1336/17 | 01.05.2017 | 80 | F | Kuwaiti |
|  | Tracheal aspirate | Kw1388/17 | 04.05.2017 | 80 | F | Kuwaiti |
| **40** | Tracheal aspirate | Kw367/17 | 26.01.2017 | 85 | M | Kuwaiti |
|  | Tracheal aspirate | Kw699/17 | 27.02.2017 | 85 | M | Kuwaiti |
|  | Tracheal aspirate | Kw1732/17 | 01.6.2017 | 85 | M | Kuwaiti |
|  | Tracheal aspirate | Kw1792/17 | 08.06.2017 | 85 | M | Kuwaiti |
|  | Tracheal aspirate | Kw1852/17 | 15.06.2017 | 85 | M | Kuwaiti |
|  | Tracheal aspirate | Kw1899/17 | 22.06.2017 | 85 | M | Kuwaiti |
| **41** | Tracheal aspirate | Kw390/17 | 29.01.2017 | 48 | M | Kuwaiti |
|  | Tracheal aspirate | Kw626/17 | 20.02.2017 | 48 | M | Kuwaiti |
| 42 | Sputum | Kw1365/17 | 02.05.2017 | 86 | M | Kuwaiti |
| **43** | Tracheal aspirate | Kw1432/17 | 09.05.2017 | 54 | M | Non-Kuwaiti |
|  | Tracheal aspirate | Kw1695/17 | 29.05.2017 | 54 | M | Non-Kuwaiti |
|  | Tracheal aspirate | Kw1825/17 | 13.06.2017 | 54 | M | Non-Kuwaiti |
|  | Tracheal aspirate | Kw1856/17 | 18.06.2017 | 54 | M | Non-Kuwaiti |
|  | Tracheal aspirate | Kw1893/17 | 22.06.2017 | 54 | M | Non-Kuwaiti |
|  | Urine | Kw1994/17 | 06.07.2017 | 54 | M | Non-Kuwaiti |
|  | Blood | Kw2074/17 | 16.07.2017 | 54 | M | Non-Kuwaiti |
| **44** | Urine | Kw1463/17 | 10.05.2017 | 79 | M | Kuwaiti |
|  | Urine | Kw1476/17 | 11.05.2017 | 79 | M | Kuwaiti |
| **45** | Tracheal aspirate | Kw1624/17 | 22.05.2017 | 62 | M | Indian |
|  | Tracheal aspirate | Kw1661/17 | 25.05.2017 | 62 | M | Indian |
| **46** | Tracheal aspirate | Kw1923/17 | 29.06.2017 | 50 | F | Kuwaiti |
|  | BAL^b^ | Kw1963/17 | 04.07.2017 | 50 | F | Kuwaiti |
|  | Tracheal aspirate | Kw1975/17 | 05.07.2017 | 50 | F | Kuwaiti |
|  | Urine | Kw2060/17 | 16.07.2017 | 50 | F | Kuwaiti |
|  | Blood | Kw2159/17 | 27.07.2017 | 50 | F | Kuwaiti |
|  | Catheter tip | Kw2472/17 | 06.09.2017 | 50 | F | Kuwaiti |
| 47 | Urine | Kw1921/17 | 29.06.2017 | 13 | M | Non-Kuwaiti |
| 48 | Blood | Kw2006/17 | 09.07.2017 | 73 | M | Kuwaiti |
| **49** | Blood | Kw2027-1/17 | 10.07.2017 | 58 | M | Non-Kuwaiti |
|  | Blood | Kw2027-2/17 | 10.07.2017 | 58 | M | Non-Kuwaiti |
| 50 | Vaginal swab | Kw2040/17 | 12.07.2017 | 73 | F | Kuwaiti |
| 51 | Urine | Kw2049/17 | 13.07.2017 | 64 | M | Non-Kuwaiti |
| 52 | Urine | Kw2058/17 | 16.07.2017 | 88 | M | Kuwaiti |
| 53 | Pleural fluid | Kw21544/17 | 25.07.2017 | 52 | F | Kuwaiti |
| **54** | Blood | Kw2260/17 | 10.08.2017 | 38 | F | Non-Kuwaiti |
|  | Urine | Kw2713/17 | 28.09.2017 | 38 | F | Non-Kuwaiti |
| **55** | Tracheal aspirate | Kw2501/17 | 07.09.2017 | 33 | F | Kuwaiti |
|  | Pleural fluid | Kw2609/17 | 18.09.2017 | 33 | F | Kuwaiti |
|  | Tracheal aspirate | Kw2647/17 | 24.09.2017 | 33 | F | Kuwaiti |
| 56 | Blood | Kw2611/17 | 18.09.2017 | 59 | M | Egyptian |

^a^Serial number of patients yielding multiple isolates are shown in Bold

^b^PEG tube swab, percutaneous endoscopic gastric tube swab; BAL, bronchoalveolar lavage

^c^M, male; F, female

^d^Non-Kuwaiti, a patient of a nationality other than official Kuwaiti national (may include Bedouins living in Kuwait)
