# Supplementary material for: Increasing prevalence, molecular characterization and antifungal drug susceptibility of serial Candida auris isolates in Kuwait
Source: PLoS One. 2018 Apr 9;13(4):e0195743. doi: 10.1371/journal.pone.0195743 (PMC5891028; doi:10.1371/journal.pone.0195743)

**Legend**

**S1 Fig. Agarose gel of PCR amplicons obtained with *C. auris*-specific (CAURF and CAURR) primers and genomic DNA from reference strains of *C. dubliniensis* (lane 1), *C. albicans* (lane 2), *C. glabrata* (lane 3)*, C. parapsilosis* (lane 3), *C. orthopsilosis* (lane 4), *C. tropicalis* (lane 5), *C. kefyr* (lane 6), *L. conglobata* (lane 7), *C. utilis* (lane 8), *C. guilliermondii* (lane 9), *C. haemulonii* (lane 10), *C. duobushaemulonii* (lane 11) and *C. auris* (lane 12).** Lane M is 100 bp DNA ladder and the positions of migration of 100 bp, 300 bp and 600 bp fragments are marked.


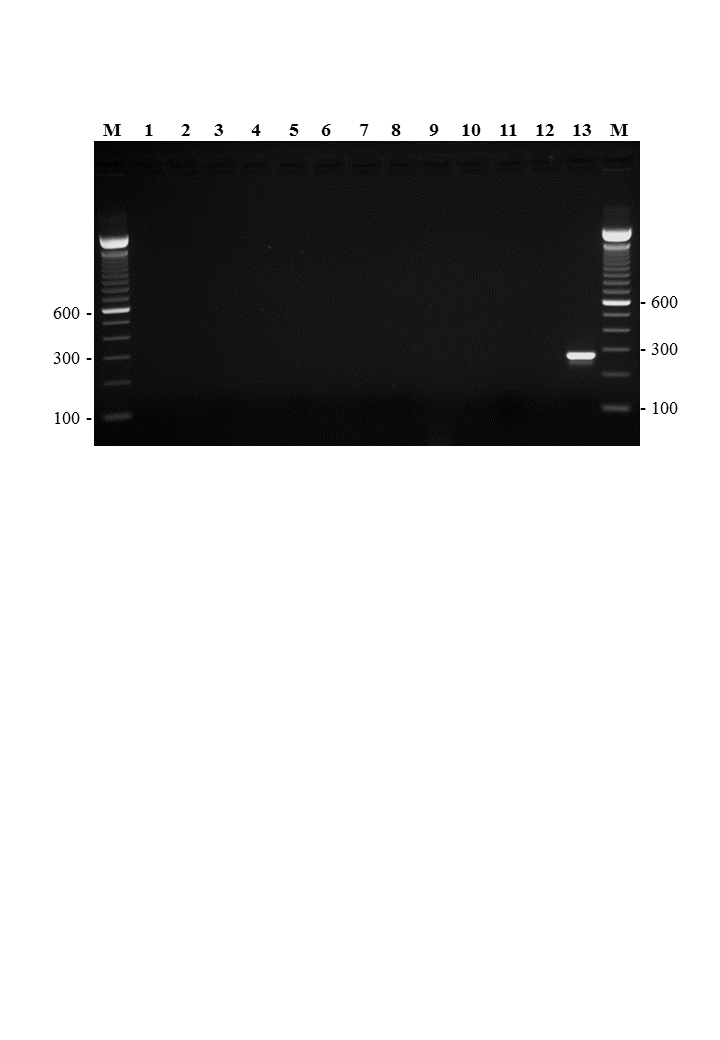

Supplement: S1 Fig — Lane M is 100 bp DNA ladder and the positions of migration of 100 bp, 300 bp and 600 bp fragments are marked. (DOCX) [file pone.0195743.s002.docx]
