# Supplementary material for: Increasing prevalence, molecular characterization and antifungal drug susceptibility of serial Candida auris isolates in Kuwait
Source: PLoS One. 2018 Apr 9;13(4):e0195743. doi: 10.1371/journal.pone.0195743 (PMC5891028; doi:10.1371/journal.pone.0195743)

**Legend**

**S2 Fig. Agarose gel of PCR products obtained during fingerprinting with minisatellite-based (M13-MIN) primer (Panel A) and microsatellite-based (GACA_4_) primer (Panel B) with genomic DNA isolated from 6 *C. auris* isolates (lanes 1-6).** Lane M is 100 bp DNA ladder and the positions of migration of 100 bp and 600 bp fragments are marked.


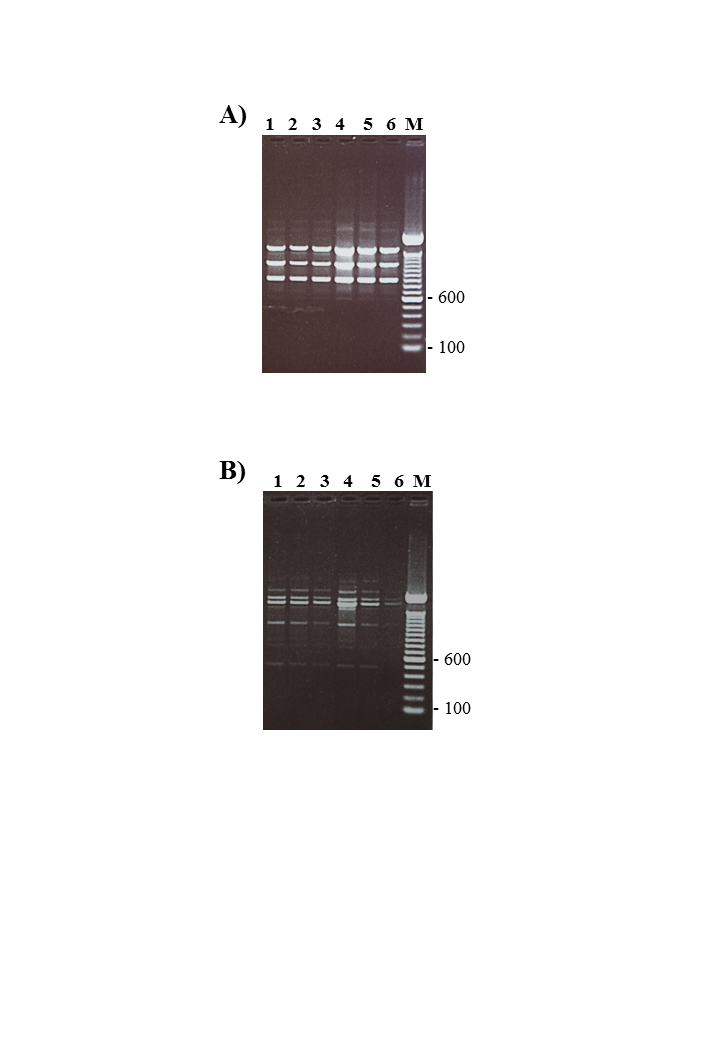

Supplement: S2 Fig — Lane M is 100 bp DNA ladder and the positions of migration of 100 bp and 600 bp fragments are marked. (DOCX) [file pone.0195743.s003.docx]
